# Ultrasensitive optoelectronic biosensor arrays based on twisted bilayer graphene superlattice

**DOI:** 10.1093/nsr/nwaf357

**Published:** 2025-08-23

**Authors:** Bowen Du, Xilin Tian, Zhi Chen, Yanqi Ge, Chuanghu Chen, Haiyan Gao, Zhongyang Liu, Jungchen Tung, Dror Fixler, Songrui Wei, Shi Chen, Han Zhang

**Affiliations:** College of Physics and Optoelectronic Engineering, Interdisciplinary Ctr High Magnet Field Phys, Shenzhen University, Shenzhen 518060; College of Physics and Optoelectronic Engineering, Interdisciplinary Ctr High Magnet Field Phys, Shenzhen University, Shenzhen 518060; Department of Biomedical Engineering, Intensive Care Unit, Shenzhen Key Laboratory of Microbiology in Genomic Modification & Editing and Application, Shenzhen Institute of Translational Medicine, Shenzhen University Medical School, Shenzhen Second People's Hospital, The First Affiliated Hospital of Shenzhen University, Shenzhen 518000; College of Physics and Optoelectronic Engineering, Interdisciplinary Ctr High Magnet Field Phys, Shenzhen University, Shenzhen 518060; College of Physics and Optoelectronic Engineering, Interdisciplinary Ctr High Magnet Field Phys, Shenzhen University, Shenzhen 518060; College of Physics and Optoelectronic Engineering, Interdisciplinary Ctr High Magnet Field Phys, Shenzhen University, Shenzhen 518060; Department of Biomedical Engineering, Intensive Care Unit, Shenzhen Key Laboratory of Microbiology in Genomic Modification & Editing and Application, Shenzhen Institute of Translational Medicine, Shenzhen University Medical School, Shenzhen Second People's Hospital, The First Affiliated Hospital of Shenzhen University, Shenzhen 518000; The Photonics Center, Shenzhen University, Shenzhen 518060; Department of Electro-Optical Engineering, Taipei University of Technology, Taipei 10608; Department of Engineering, Bar-Ilan University, Ramat Gan 52001; College of Physics and Optoelectronic Engineering, Interdisciplinary Ctr High Magnet Field Phys, Shenzhen University, Shenzhen 518060; Department of Biomedical Engineering, Intensive Care Unit, Shenzhen Key Laboratory of Microbiology in Genomic Modification & Editing and Application, Shenzhen Institute of Translational Medicine, Shenzhen University Medical School, Shenzhen Second People's Hospital, The First Affiliated Hospital of Shenzhen University, Shenzhen 518000; College of Physics and Optoelectronic Engineering, Interdisciplinary Ctr High Magnet Field Phys, Shenzhen University, Shenzhen 518060

**Keywords:** biosensor, twisted graphene, two-dimensional material, local surface, plasmon resonance, CRISPR

## Abstract

Recent advances in twistronics have revealed tunable optoelectronic properties in twisted bilayer graphene (tBLG), including angle-dependent dielectric responses and enhanced light absorption due to van Hove singularity (VHS). However, achieving high photoresponsivity in tBLG-based sensors typically requires intense illumination. We present an ultrasensitive optoelectronic biosensor integrating tBLG superlattices with Au nanodisks and clustered regularly interspaced short palindromic repeat (CRISPR)-Cas12a via DNA origami. By aligning the 9.4° tBLG's VHS absorption spectrum with Au nanodisks’ plasmonic resonance at 60 μW, we achieve a 7-fold photocurrent enhancement over pristine tBLG. CRISPR-Cas12a-mediated trans-cleavage dynamically modulates the local dielectric environment, enabling sub-femtomolar (44.63 attomolar, aM) nucleic acid detection without external amplification. Clinical validation using lung cancer samples shows high concordance with quantitative polymerase chain reaction (qPCR), demonstrating real-time, label-free detection of microRNA (miRNA). This hybrid platform combines moiré-engineered optoelectronics with programmable bio-nanoarrays, offering a scalable solution for precision diagnostics with ultralow detection limits and rapid response times.

## Teaser

This study pioneers an opto-electronic biosensor by synergizing twist-engineered moiré superlattices in graphene with CRISPR technology, enabling sub-femtomolar nucleic acid detection under low-light conditions

## INTRODUCTION

Moiré superlattices, arising from rotational or translational mismatches between stacked periodic structures [1–3], have revolutionized the manipulation of electronic properties in two-dimensional (2D) materials. These tunable quantum platforms enable exotic phenomena, including superconductivity [4–6], Mott insulators [7] and quantum Hall effects [8,9]. Notably, twisted bilayer graphene (tBLG) superlattices, engineered through interlayer rotation, exhibit angle-dependent bandgap modulation [10,11], topological valley transport [12,13] and a moiré pattern [14,15], offering unprecedented design flexibility for optoelectronic devices. For large twist angles (>5°), tBLG preserves monolayer-like linear band dispersion near the Dirac point [16], while intersecting Dirac cones generate saddle points in the density of states (DOS), creating van Hove singularity (VHS) that enhances optical absorption and Raman resonance [17,18]. Crucially, the twist angle (*θ*) governs electronic coupling between atomic layers [19], enabling *θ*-tunable dielectric responses to external electric fields [20]. This unique angle-electric field interplay establishes tBLG as a promising candidate for moiré-enhanced optoelectronic sensors, where superlattice-specific dielectric constants can be precisely optimized through *θ*-selection.

Although tBLG demonstrates superior optical absorption compared to monolayer graphene, realizing high photoresponsivity and selectivity in practical devices remains non-trivial. Despite theoretical predictions that VHS energy alignment with incident photon energies could enable photocurrent generation [21], experimentally achieving microampere-level photocurrents typically requires intense light irradiation (>1000 μW) [22]. To overcome this limitation, nanostructuring has been explored to enhance light-matter interactions in 2D materials [23], giving rise to non-linear phenomena, such as trion excitons [24], polaritons [25] and Rabi splitting [26]. Given tBLG's intrinsic sub-micrometer exciton transport length and Bohr radius, confining polaritons within tailored nanoarrays represents a viable strategy to engineer localized density of states (LDOS) near material interfaces [27]. Such LDOS enhancement can facilitate strong exciton–plasmon coupling modes [28], which are critical for optoelectronic device operation. What's more, the integration of external resonators for mode engineering may perturb structural resonances, presenting a new mechanism for innovative biosensing applications.

Recently, significant advancements in biosensing have been driven by breakthroughs in materials science and sensing paradigm development [29–31]. Among optical detection techniques, surface plasmon resonance (SPR) sensors remain widely adopted due to their label-free, real-time detection capabilities [32]. These systems exploit refractive index changes at metal–dielectric interfaces to detect biomolecular interactions [33], which restricts their use in ultrasensitive assays. Although nanomaterial-integrated SPR sensors have achieved partial improvements through localized field enhancement, purely optical approaches fall short of the precise sub-femtomolar detection threshold required for clinical diagnostics.

Here, we present a hybrid optoelectronic biosensor integrating Au nanodisks/tBLG heteroarrays with clustered regularly interspaced short palindromic repeat (CRISPR)-Cas12a via DNA origami technology. Unlike conventional optical methods, this platform converts optical signals into electrical outputs through exciton–plasmon coupling in the Au nanodisks/tBLG interface, enabling ultrasensitive detection under low-light conditions. By precisely aligning the VHS absorption spectrum of tBLG with the plasmonic resonance of Au nanodisks positioned on the tBLG surface, we establish coupling modes that amplify optical transduction efficiency. This design enables amplification-free nucleic acid detection with femtomolar sensitivity, as demonstrated by the detection of single-stranded DNA at concentrations down to sub-femtomolar, exceeding traditional DNA biosensors by four orders of magnitude. Our work establishes a general framework for moiré-engineered optoelectronics, combining twist-angle-tuned dielectric properties with CRISPR-mediated specificity for multiplexed biosensing applications.

## RESULTS

The CRISPR-Cas12a system, a revolutionary genome-editing tool, has found extensive applications in biosensing through its exceptional single-base recognition capability. By guiding Cas12a to target specific nucleic acid sequences with near-perfect complementarity, this system enables trans-cleavage of DNA strands with high specificity. Our previous work demonstrated the integration of CRISPR-Cas12a with SPR sensors, achieving label-free detection without the need for complex sample preparation or amplification. However, conventional SPR-based approaches utilizing single-stranded DNA (ssDNA) probes face critical limitations: entanglement between adjacent probes disrupts probe–target interactions, while uncontrolled distances between probes and the sensor surface lead to non-functional probe occupation [34]. DNA nanotechnology offers a promising solution through self-assembled 3D structures, such as scaffolded DNA origami [35]. By exploiting Watson–Crick base pairing, this method enables precise control over nanoscale architectures [36]. In our work, tetrahedral DNA origami templates allow for deterministic positioning of Au nanoparticles (AuNPs) with nanometer accuracy, ensuring uniform probe heights and optimal spacing between functional elements. This level of spatial regulation not only mitigates the above-mentioned challenges but also provides a scalable platform for multiplexed biosensing applications.

**Figure 1.**
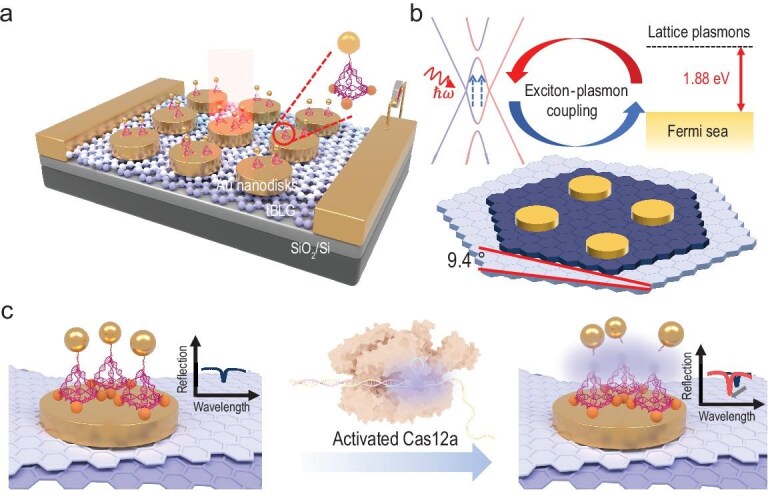
Construction of the structure. (a) Schematic diagram depicting the heterostructure composed of AuNPs, DNA origami, Au nanodisks and tBLG. (b) Illustration of the principle of exciton−plasmon coupling. (c) Illustration of the principle for miRNA-21 detection using the CRISPR-Cas12a system.

In our experiment, the schematic diagram of optoelectronic biosensor arrays in tBLG is illustrated in Fig. 1a. High-quality monolayer graphene flakes were transferred onto silicon dioxide substrates and subjected to femtosecond laser cutting to form tBLG with a twist angle of 9.4°, yielding a moiré superlattice with a lattice constant of 1.501 nm. This large twist angle was chosen to minimize fabrication defects observed in smaller twist angles (<5°). Au nanodisks were fabricated by patterning the polymethyl methacrylate (PMMA) spacer using electron-beam lithography (EBL) on the surface of tBLG. We designed the mesa structures with a resonant wavelength that matches the VHS absorption to ensure an exciton–photon coupling, as shown in Fig. 1b. For biosensor integration, tetrahedral DNA origami–AuNP conjugates were immobilized on the Au nanodisk arrays. The DNA origami framework ensured precise control over AuNP positioning, maintaining a uniform inter-particle distance between AuNPs and both the Au nanodisks and neighboring AuNPs. This configuration is critical for optimizing the local dielectric environment and sustaining efficient exciton–plasmon coupling modes. All the processes are described in the Methods and Fig. S1. In this study, the sensor operated through CRISPR-Cas12a-mediated trans-cleavage. In the absence of target DNA, the DNA origami-encapsulated AuNPs disrupted the plasmonic coupling between the Au nanodisk array and tBLG, leading to destruction of resonance and a reduction in photocurrent, as shown in Fig. 1c. Upon target DNA recognition, Cas12a trans-cleaved the ssDNA linker within the DNA origami, releasing the AuNPs and restoring the original dielectric environment. This process reactivated the exciton–plasmon coupling mode, as evidenced by the recovery of the resonance peak position and photocurrent signal. This phenomenon was illustrated in the simulated reflection spectra for both configurations with and without AuNPs structures, utilizing the finite-difference time-domain (FDTD) method.

**Figure 2.**
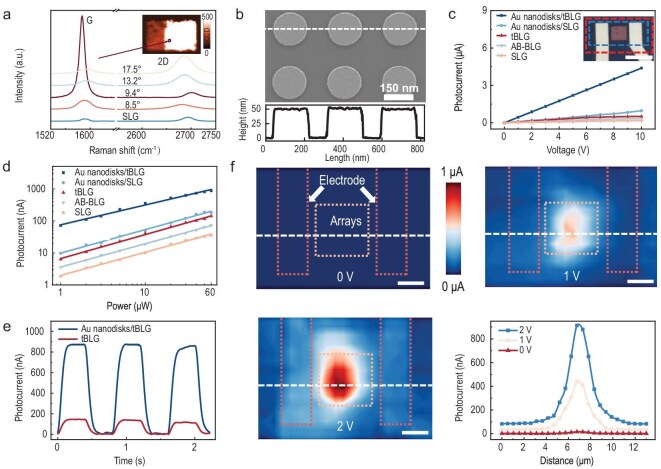
Selective enhancement of tBLG structures via Au nanodisks. (a) The Raman spectra of SLG and tBLG domains with twist angles of 8.5°, 9.4°, 13.2° and 17.5° were analyzed. The inset image shows a G-band intensity mapping of the 9.4° tBLG domain. (b) The top image shows the SEM image of Au nanodisks. The bottom image displays a height map with white dashed lines. (c) The variation of photocurrents with bias voltage for SLG, AB-stacked bilayer graphene (AB-BLG), Au nanodisks/SLG, tBLG and Au nanodisks/tBLG. Inset, the red and blue boxes represent the tBLG area and the photocurrent mapping area, respectively. The power is 60 μW. Scale bar is 10 μm. (d) Photocurrents generated from SLG, AB-BLG, Au nanodisks/SLG, tBLG and Au nanodisks/tBLG were measured as a function of incident power, with a bias voltage of 2 V. (e) Real-time switching behavior under conditions of a laser wavelength of 660 nm, a power of 60 μW and a bias voltage of 2 V. (f) Photocurrents of the scanning Au nanodisks/tBLG structure are shown at bias voltages of 2 V, 1 V and 0 V. The line-scanning distribution of the Au nanodisks/tBLG structure. Red dashed lines demarcate electrode contact areas, golden dashed lines outline Au nanodisk array regions and white dashed lines indicate line-scanning trajectories.

The pronounced upshift in the Raman G-band of tBLG served as a definitive signature of VHS and enhanced interlayer coupling. Figure 2a illustrates the Raman spectra of single-layer graphene (SLG) and tBLG with twist angles of 8.5°, 9.4°, 13.2° and 17.2°, recorded under 633 nm laser excitation. It was observed that the G-band intensity peaked at a twist angle of 9.4° tBLG, whereas the 2D-band intensity increased steadily with larger twist angles. As shown in Fig. S2, the I_G_/I_2D_ ratio for the 9.4° tBLG was approximately ∼28.11-fold higher than that of SLG. Additionally, spatial Raman mapping over the 9.4° tBLG region indicated uniform intensity distributions for both spectral features, verifying the structural consistency at the micrometer scale. The scanning electron microscopy (SEM) image of the Au nanodisks/tBLG heterostructure depicted a periodic arrangement of 50 nm-thick Au nanodisks with 274 nm periodicity, forming a 7 μm × 7 μm heterojunction area (Fig. 2b). The height profile, indicated by white dashed lines at the bottom, was obtained using an atomic force microscope (AFM).

The light–matter interaction in the tBLG of VHS was enhanced by Au nanodisks, which can create the coupling mode between excitons and plasmons, thereby increasing the generation of photocurrent when illuminated. For the energy of the VHS, we extracted an estimation of the twist angle *θ* considering the correlation E_vhs_ = E_0_*|sin3*θ|, where E_0_ = 3.9 eV. This formula is derived from fitting the resonance transition energy as a function of *θ*, based on the results from the calculations. Given that the energy interval of the two VHSs in the 9.4° domain aligned with both the incident photon energy and the resonance peak of Au nanodisks at 1.88 eV, an enhanced photocurrent was observed (Fig. 2c). Current–voltage measurements revealed that the Au nanodisks/tBLG heterojunction achieved higher photocurrent density and superior bias-modulation sensitivity compared to pristine tBLG, originating from the enhanced coupling efficiency. Quantitative analysis of photoresponsivity showed that both heterojunction and bulk domains exhibited photocurrent increases with incident power from 1 to 60 μW (Fig. 2d). Notably, the heterojunction achieves a record photoresponsivity of 14.64 mA/W, which exceeds the intrinsic tBLG value (2.34 mA/W) by a factor of 6.27 while also outperforming other stacked or SLG structures. This enhanced responsivity corresponds to an improvement in external quantum efficiency (EQE) to 27.51%, indicating enhanced photon-to-electron conversion capability, as shown in Table S1. Real-time switching behavior confirmed the heterojunction's exceptional stability with negligible signal decay over multiple light/dark cycles (<5%) (Fig. 2e).

Additionally, we further conducted the spatially resolved photocurrent mapping under 660 nm illumination (8 μm spot size), revealing a spatially resolved photocurrent distribution (Fig. 2f). The maximum photocurrent density (under a 2 V bias) occurred at the heterojunction center, reaching seven times the value of the 9.4° pristine tBLG. The line-scanning profile displayed a Gaussian-like distribution with a full-width-at-half-maximum (FWHM) of ∼1.5 μm, consistent with the plasmonic confinement by the Au nanodisks. Furthermore, we conducted Raman mapping and photocurrent imaging of the active-region tBLG structure to demonstrate uniform response distribution across the active area, as shown in Fig. S3. Such spatially resolved optoelectronic behavior not only validates the device architecture but also provides a pathway for ​submicron-scale biosensing​ through precise control of plasmonic hotspots and CRISPR-mediated dielectric switching.

**Figure 3.**
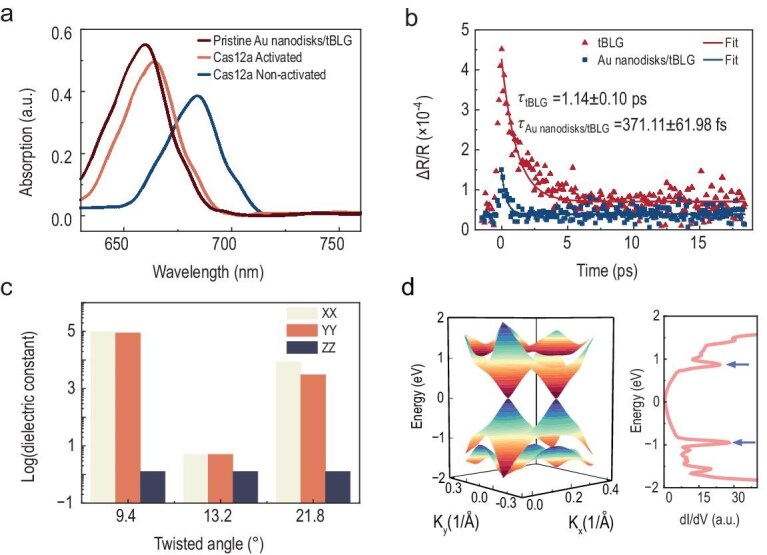
Optical spectra and simulation results. (a) Comparison of absorption spectra for Pristine Au nanodisks/tBLG, Cas12a-activated AuNPs/Au nanodisks/tBLG system and the corresponding non-activated control. (b) Characterization of tBLG and Au nanodisks/tBLG structures via pump-probe spectroscopy. (c) Simulated dielectric constants of 9.4°, 13.2° and 21.8° tBLG structures. (d) Left: the calculated energy band structure of 9.4° tBLG; right: spatially resolved dI/dV by STM/STS. Blue arrows designate VHS with characteristic energy separation 2E_VHS_ ≈ 1.84 eV.

The absorption spectrum shown in Fig. 3a revealed a distinct spectral shift between the Cas12a-activated AuNPs/Au nanodisks/tBLG system and its non-activated control counterpart. This observed shift originated from the restoration of exciton–plasmon coupling following the release of AuNPs from the DNA origami framework. Initially, DNA-anchored AuNPs disrupted the intrinsic exciton–plasmon coupling between tBLG and Au nanodisks, resulting in a red-shifted absorption peak at 684 nm. Upon Cas12a-mediated trans-cleavage, AuNPs detached, re-establishing efficient coupling and driving the absorption band to blue-shift to 663 nm. Notably, the Cas12a-activated Au nanodisks/tBLG sample exhibited a red-shifted absorption peak at 663 nm (compared to 660 nm for the pristine Au nanodisks/tBLG system) with significantly reduced intensity. This was attributed to residual DNA strand retention at the Au-tBLG interface post-trans-cleavage, which modulates the local dielectric environment and consequently disrupts exciton–plasmon coupling efficiency. Time-resolved differential reflection measurements were conducted to investigate the electron dynamics of excitons in excited states during interband transitions in the Au nanodisks/tBLG coupling. As shown in Fig. 3b, the ΔR/R signal decayed exponentially for both Au nanodisks/tBLG domains (squares) and pure tBLG (dots). Fitting the experimental data with a single-exponential function, I(t) = A_0_ exp(−t/τ), the extracted decay time constants are 371.11 ± 61.98 fs and 1.14 ± 0.10 ps for the two samples, respectively. The ΔR/R dynamics of pure tBLG strongly depend on the transition energy, showing long decay times due to bound excitons. Supplementary femtosecond pump-probe experiments compared carrier relaxation dynamics of Au–SLG, tBLG and Au–tBLG (Fig. S4). Au–SLG shortens relaxation time by 35.5% (555.62 fs vs SLG's 861.44 fs), indicating Au nanodisks accelerate recombination via interfacial scattering/hot electrons but yield limited photoresponsivity, ruling out dominant plasmonic contributions. In contrast, tBLG prolongs relaxation time by 32.3% (1.14 ps vs SLG), ascribed to moiré-induced VHS states that localize carriers and dominate the photoresponse. Notably, Au–tBLG further shortens relaxation to 371 fs (3-fold faster than tBLG) while achieving 6.27-fold higher photoresponsivity. Collectively, the incorporation of such nanostructures enhances light–matter interaction, converting ground-state electrons to high-energy coupled polaritons and accelerating decay, whereas Au nanodisks provide auxiliary gains through light-field regulation and optimized carrier transport.

Next, we systematically investigated the twist-angle-dependent band engineering of tBLG heterostructures. Angular displacement between the moiré superlattice layers will induce Dirac cone splitting with an angular-dependent energy shift. This angular defect significantly modifies the Dirac band structure through interlayer coupling, giving rise to novel electronic features. These saddle points manifest as VHS in DOS. Band structure calculations for tBLGs were performed based on density functional theory (DFT), as shown in Fig. S5. We then calculated the dielectric constant of certain *θ* tBLG by using density functional perturbation theory (DFPT), only when the resulting unit cell is sufficiently small, as illustrated for the tBLG structures with angles of 9.43°, 13.2° and 21.8° (Fig. 3c). Notably, the in-plane (XX/YY) dielectric constants of 9.4° tBLG exhibited values orders of magnitude higher than those at θ = 13.2° and 21.8°, whereas the out-of-plane (ZZ) dielectric constant remained low across all twist angles. This anisotropic behavior implies that effective transverse electric field modulation of tBLG devices is feasible only through in-plane polarization. The superior performance of the 9.4°-twisted tBLG arises from its exceptionally high in-plane dielectric constant (ε_XX_/ε_YY_), which significantly enhances charge screening and confines electric fields more effectively at the Au nanodisks/tBLG interface. This enhanced field confinement amplifies plasmonic ‘hot spots’ and promotes exciton generation.

Therefore, precise control of the twist angle is of utmost importance. To experimentally verify the twist angles, we presented selected area electron diffraction (SAED) patterns of all tBLG domains (Fig. S6). Additionally, SAED patterns from multiple regions were provided in Fig. S7 to confirm the crystallinity and uniformity of the tBLG layers. For non-negligible *θ*, the tBLG electronic spectrum evolves into four Dirac cones inherited from individual monolayers, which intersect to form saddle points in the reciprocal space. Moreover, scanning tunneling spectroscopy (STS) measurement of the LDOS can conclusively verify the VHS energy of 9.4° tBLG. As shown in Fig. 3d, this LDOS peak was observed at an energy level of approximately 1.84 eV. This value matches our simulated VHS energy for 9.4° tBLG and closely aligns with the 660 nm photon energy (1.88 eV).

**Figure 4.**
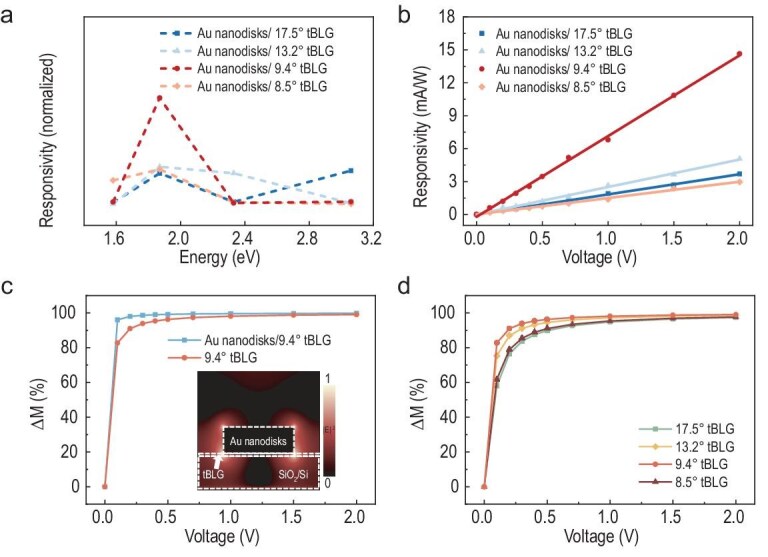
Variation of photocurrent and modulation depth of Au nanodisks/tBLG heterostructure at different twist angles. (a) The photocurrent versus the energy of the incident photon. Au nanodisks/tBLG domains with twist angles of 8.5°, 9.4°, 13.2° and 17.5°. The plots are normalized with those of AB-BLG. (b) Variations of responsivity for Au nanodisks/tBLG structure at 8.5°, 9.4°, 13.2° and 17.5°, respectively. (c) The modulation depth of the 9.4° twist angle Au nanodisks/tBLG structure varies with the bias voltage. Inset: the simulation of light field distribution for the Au nanodisk structure with 660 nm incident light. (d) The modulation depth of tBLG structures varies with the bias voltage at 8.5°, 9.4°, 13.2° and 17.5°, respectively.

Previous discussions have suggested that the formation of VHS is central to the mechanism of exciton–plasmon coupling in Au nanodisks/tBLG heterostructure systems, which is significantly influenced by the twist angle. Empirical observations revealed a direct correlation between the photocurrent characteristics and the VHS energy levels as a function of incident photon energy, as demonstrated in Fig. 4a. For systematic comparison, we fabricated devices with twist angles of 8.5°, 9.4°, 13.2° and 17.5°, all integrated with Au nanodisks of identical size (resonant at 660 nm). The energy interval of the VHS (2*E_VHS_*) in the 9.4° tBLG domain was determined to be 1.84 eV, showing exceptional alignment with the incident photon energy (1.88 eV) at 660 nm wavelength. This spectral coincidence enables enhanced light–matter interaction, leading to an ∼2-fold photocurrent enhancement at 2*E_VHS_* compared to other devices excited near analogous VHS energy levels. As shown in Fig. 4b, all tBLG devices exhibited significant responsivity shifts under bias modulation. Notably, the 9.4° tBLG/Au heterojunction demonstrated the most pronounced bias-dependent response, achieving near-complete modulation depth (*ΔM* ≈ 100%) at 0.2 V. The modulation depth is given by the formula *ΔM* *=* (*C_p_−C*)/(*C_p_ + C*), where *C_p_* and *C* represent source-drain currents of the device at varying bias voltages and 0 V bias voltage, respectively. Finite-difference time-domain simulations of the coupling mode revealed localized ‘hot spots’ at the heterojunction interface (inset in Fig. 4c). Furthermore, the threshold voltage for photoresponsivity in pure tBLG devices was analyzed (Fig. 4d). The 9.4° tBLG sample exhibited significantly lower thresholds compared to other angles, consistent with the larger in-plane dielectric constant at this twist angle (Fig. 3c).

**Figure 5.**
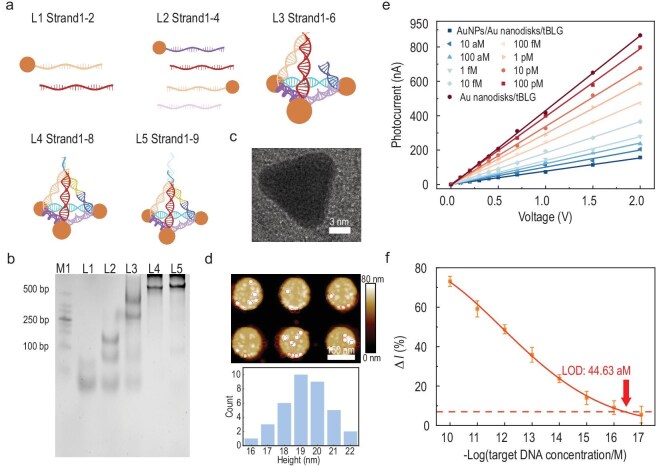
Detection of miRNA through the Au nanodisks/tBLG heterostructure. (a) Schematic representation of DNA origami assembly. (b) PAGE of the DNA origami folding process. (c) TEM image of DNA origami formed by 1–8 strands. (d) Top: AFM image of the AuNPs/origami/Au nanodisks heterostructure. Bottom: height profile of AuNPs/DNA origami attached to Au nanodisks. (e) The curves depict the photocurrent variations with bias voltage for T-DNA at concentrations ranging from 100 pM to 1 fM. (f) Correlation between the miRNA-21 target DNA concentration (10 aM–100 pM) and *ΔI* signal.

Finally, we translated this mechanism into a highly sensitive biological detection platform for human tumor markers. By integrating CRISPR-Cas12a with the Au nanodisks/tBLG heterostructure, we achieved single-base resolution detection of microRNA (miRNA)-21 at ultra-low concentrations. The trans-cleavage efficiency of CRISPR RNA (crRNA)–target DNA (T-DNA) complexes was validated through polyacrylamide gel electrophoresis (PAGE) and SHERLOCK assays (Fig. S8). The biosensor with Au nanodisks/tBLG heterostructure arrays was constructed for the bio-detection of the tumor marker miRNA-21 by utilizing the DNA origami structure. DNA origami folding was performed as illustrated in Fig. 5a. The binding of single strand 9 to the DNA origami consisting of strands 1–8 is crucial because strand 9 introduces the trans-cutting site containing the Cas12a protein and a poly(A) tail as the anchoring site for the Au nanoparticles. By combining AuNPs with DNA origami via strand 9, the AuNPs/DNA origami structure is obtained as a probe that is subsequently fixed to the sensor. The folded products were collected and subjected to PAGE, as shown in Fig. 5b. The reactants from different DNA strands were added into the L1–L5 channels, respectively, allowing for the determination of specific structural integrity and high yield. After optimizing the protocol (in Methods), a clear triangle was observed under a transmission electron microscope (TEM), as shown in Fig. 5c, indicating the successful assembly of DNA origami. The AuNPs/DNA origami/Au nanodisks/tBLG heterostructure arrays were then examined by AFM to observe the height of the probe, as shown in Fig. 5d. The attachment of the probe to the surface of the Au nanodisks and the small size of the combined probe were visible. Most of the probe heights are between 18 and 21 nm with high flatness, which is the basis for our accurate FDTD simulations.

For biosensor characterization, we quantified the photocurrent response to T-DNA concentrations spanning 100 pM to 10 aM under optimized conditions in Fig. 5e. Signal analysis involved three stages: baseline (*I₀*), post-AuNP immobilization (*I₁*) and post-trans-cleavage (*I₂*). The signal change ratio was calculated as: Δ*I* = (*I*_2_−*I*_1_)/*I*_0_ × 100%. Threshold determination followed IUPAC guidelines, incorporating blank measurements and 3σ standard deviation, yielding a threshold value of 6.45%. This enabled the limit of detection (LOD) calculation as 44.63 aM, validated through a 10 aM–100 pM concentration gradient (Fig. 5f). Real-time kinetic analysis of CRISPR-Cas12a-mediated AuNP release (Fig. S9) further demonstrated the platform's operational stability under dynamically evolving biointerfacial conditions.

Compared to conventional methods (Fig. S10 and Table S2), our hybrid system demonstrates superior performance: 44.63 aM LOD, 1 h detection time and signal amplification without external pre-amplification. This enhancement arises from two synergistic mechanisms: Au nanodisks structural resonance and tBLG bandgap alignment, both exhibiting exceptional sensitivity to environmental perturbations. The integrated photonic–electronic transduction pathway enables real-time, label-free detection of nucleic acid biomarkers with sub-femtomolar sensitivity.

**Figure 6.**
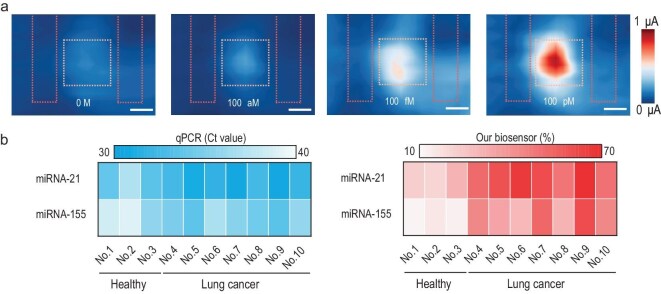
The spatially resolved photocurrent mapping of Au nanodisks/tBLG linked with AuNPs following trans-cleavage, and clinical samples validation. (a) These images present the photocurrent mapping of the Au nanodisks/tBLG structure linked with AuNPs after introducing 0 M, 100 aM, 100 fM and 100 pM concentrations of target DNA, respectively. (b) Comparison of samples collected from healthy individuals and lung cancer patients. The samples were measured using both qPCR and biosensor methods to evaluate their performance.

The spatially resolved photocurrent mapping of the AuNPs/Au nanodisks/tBLG heterostructure revealed distinct current distributions upon T-DNA introduction (Fig. 6a). Pump-probe measurements before and after analyte addition are shown in Fig. S11, demonstrating the dynamic response characteristics. Fluorescence quenching assays demonstrated that crRNA–T-DNA recognition strictly requires perfect complementarity, with significant fluorescence enhancement exclusively observed in matched pairs (Fig. S12). This stringent specificity confirms the system's ability to discriminate single-nucleotide mismatches. The precisely engineered 3D DNA origami structure plays a critical role in modulating the charge transport pathways through the heterojunction interface. This methodology was validated using clinical lung cancer samples (Fig. 6b), yielding results with excellent concordance to quantitative polymerase chain reaction (qPCR) analysis. And the biosensor achieved ultra-sensitive miRNA detection at femtomolar concentrations, exhibiting significantly higher signal variation compared to qPCR measurements. Remarkably, the device still retained high response fidelities after 20 day incubation in PBS buffer and whole blood, as confirmed through target DNA challenge tests, indicating exceptional long-term stability for clinical applications (Fig. S13). Moreover, the sensor demonstrated exceptional selectivity by accurately distinguishing between different miRNA species through CRISPR-Cas12a editing. Collectively, these findings establish this hybrid platform as a robust, label-free diagnostic tool with high clinical translational potential, offering real-time detection of biomarkers in complex biological matrices.

## DISCUSSION

This study establishes a paradigm for ultrasensitive biosensing through synergistic integration of twist-engineered tBLG, Au nanodisks and CRISPR-Cas12a. The angle-tuned VHSs in tBLG provide a natural pathway for low-light photocurrent generation, while DNA origami ensures precise positioning of functional elements for optimal signal transduction. The sensor's sub-femtomolar detection limit, rapid response (<1 h) and multiplexing capability surpass conventional methods by four orders of magnitude. By linking CRISPR's specificity with tBLG's unique electronic properties, we achieved label-free, real-time detection of biomarkers in complex biological matrices. This work not only advances moiré superlattice-based optoelectronics but also paves the way for next-generation clinical diagnostics with unparalleled sensitivity and specificity.

This work pioneers an ultrasensitive optoelectronic biosensing platform by synergistically integrating tBLG superlattices, plasmonic nanoarrays and CRISPR-Cas12a technology. Through moiré-engineered dielectric engineering and exciton–plasmon coupling, we achieved a record-breaking photoresponsivity of 14.64 mA/W and sub-femtomolar nucleic acid detection sensitivity (44.63 aM) under low-light conditions (60 μW). The DNA origami-mediated dynamic modulation of the local dielectric environment enabled label-free, real-time detection of miRNA-21 in clinical lung cancer samples, demonstrating exceptional agreement with qPCR. This hybrid platform transcends traditional optical and electronic sensing paradigms by merging twist-angle-tunable electronic properties with programmable bio–nano interfaces. The convergence of CRISPR's molecular specificity and tBLG's moiré-enhanced light–matter interactions creates a scalable framework for multiplexed biodetection, addressing critical challenges in ultrascale diagnostics. Beyond nucleic acid sensing, this strategy holds transformative potential for detecting proteins, exosomes and other biomarkers in complex biological matrices. By pushing the boundaries of sensitivity, speed and versatility, our work charts a roadmap for next-generation precision medicine tools, where nanoscale materials and genome-editing technologies converge to unlock unprecedented diagnostic capabilities.

## MATERIALS AND METHODS

### Device fabrication

Polymethyl methacrylate (Allresist, ARP 672.45) was spin-coated onto graphene single crystals grown via chemical vapor deposition (CVD) on copper foil. The PMMA/graphene/copper stack was cut with an 800 nm, femtosecond laser (Spectra Physics), followed by copper foil etching. After PMMA removal in acetone, the graphene/SiO_2_/Si substrate was placed on a high-precision rotation stage as the tBLG bottom layer. Another PMMA/graphene layer was transferred to a PDMS-coated glass slide to form a glass/PDMS/PMMA/graphene stack. The PMMA/tBLG/SiO₂/Si sample was prepared by rotating the bottom graphene (*θ* twist angle) and adhering the top graphene via a 3D translation stage. Cr/Au electrodes (10/40 nm) and nanodisks (5/45 nm) were fabricated via EBL (EBPG 5150) and evaporation. Details are shown in Fig. S1.

### DFT simulation

All calculations are performed based on DFT. The generalized gradient approximation (GGA) with the Perdew–Burke–Ernzerhof (PBE) functional is utilized to describe the exchange–correlation interaction [37,38]. The plane-wave energy cutoff is 500 eV. A 2 × 2 × 1 Monkhorst–Pack K-mesh is set to sample the Brillouin zone integration. To avoid the interaction between adjacent layers, a vacuum of 15 Å is created along the *z*-axis [39]. In the process of structural relaxation, the force convergence of 0.01 eV/Å and the energy convergence criteria of 10^−5^ eV are set to ensure that atomic sites and lattice constants are fully relaxed. The total energy convergence criterion of the self-consistent field calculation is 1 × 10^−7^ eV. The DFT-D3 method of Grimme is considered to correct the van der Waals interaction [40]. The dielectric constant is calculated by the DFPT [41] and can be found in the OUTCAR file.

### Electrical measurements

Electrical measurements were conducted using a lock-in amplifier (CIQTEK Melab), while photoelectrical measurements employed a scanning photocurrent microscope. Lasers (405, 532, 660, 785 nm) with adjustable frequencies were used. The output frequency was set to 1 kHz, and beams were focused onto the device via a ×100 objective lens (1 μm spot size). The device was connected to a pre-amplifier and ITECH IT2805 voltage source for bias supply, with photocurrents measured by the lock-in amplifier. During laser scanning, photocurrents and beam positions were simultaneously recorded via computer for mapping. All measurements were performed under ambient conditions.

### Target DNA detection on device

The Au nanodisks/tBLG/heterostructure was rinsed with Tris-HCl/magnesium sulfate (TM) buffer and dried with N₂. The probe was incubated for 30 min for DNA origami assembly, followed by TM buffer washing. Strand 9 was introduced (30 min), then blocked with MCH (15 min), and the AuNPs solution was applied (30 min). After rinsing and drying, AuNPs/DNA origami probes were formed for photocurrent measurement. CRISPR reactions were incubated on the flow cell at 37°C for 30 min. Non-specific binding was removed via proteinase K (10 min) after TM buffer rinsing. Signals were recorded post-final rinse. The synthesis sequence of DNA origami, and the synthesis sequences for crRNA and target DNA can be found in Table S3.

### Clinical samples

miRNA was extracted and purified from plasma samples of lung cancer patients using a miRNA extraction kit (Yuanye R33050), purified and stored at −80°C. Pre-determined cycle threshold (Ct) values for target genes were provided by Shenzhen Second People's Hospital. Ethical approval (2024-495-01PJ) and compliance with ICH-GCP, the Declaration of Helsinki, and institutional guidelines were confirmed.

### Statistics

The data are displayed as mean ± standard deviation; all measurements were performed in three parallel experiments (*n* = 3). Correlations were performed with linear regression to determine the goodness of fit (Pearson's correlation coefficient, R^2^). For inter-sample comparisons, multiple pairs of samples were analyzed using a two-tailed *t*-test. The resulting *P* values were adjusted for multiple hypothesis testing using Bonferroni correction. *P < 0.05, **P < 0.01 and ***P < 0.001 indicate obvious statistical differences. All statistical analyses were performed using OriginPro.

## Supplementary Material

nwaf357_Supplemental_Files

## FUNIDNG

This work was supported by the National Key Research and Development Plan of China (2024YFB2808100 and 2022YFA0912500), the National Natural Science Foundation of China (62305229 and 32220103001), the High-Level Talent Innovation and Entrepreneurship Plan of Shenzhen (JSGGKQTD20221101115657034), the Department of Education of Guangdong Province (2022ZDZX1023), the Shenzhen Natural Science Fund (the Stable Support Plan Program 20220810161836001), the Shenzhen Key Laboratory of Microbiology in Genomic Modification & Editing and Application (Shenzhen Science and Technology Program, ZDSYS20230626090759006), the Taipei University of Technology-Shenzhen University Joint Research Program (2023016) and the LingChuang Research Project of China National Nuclear Corporation (CNNC-LCKY-202268).
