# Supplementary material for: Ultrasensitive optoelectronic biosensor arrays based on twisted bilayer graphene superlattice
Source: Natl Sci Rev. 2025 Aug 23;12(10):nwaf357. doi: 10.1093/nsr/nwaf357 (PMC12492000; doi:10.1093/nsr/nwaf357)
Supplement: nwaf357_Supplemental_Files [file nwaf357_supplemental_files.zip › Supplementary data.pdf]

# Supplementary Information

# Sub-Attomolar-Level Biosensing of Cancer Biomarkers Using SHG Modulation in DNA-Programmable Quantum Dots/$MoS_2$ Disordered Metasurfaces

**Bowen Du *et al.***

**This PDF file includes:**

**Supplementary Text**

**Figure. S1 to S7**

**Table S1 to S2**

## Supplementary Text

### Characterization

In this study, a multimodal characterization platform was used for systematic analysis of the samples: The microscopic morphological features were observed using an Olympus BX53 upright fluorescence microscope for bright-field and dark-field dual-mode imaging, acquiring high-resolution digital images. The nanoscale structure was analyzed using a field emission transmission electron microscope (TEM, JEOL JEM-F200), which performed DNA origami structure characterization at an accelerating voltage of 200 kV. The surface morphology of the structure was examined using an atomic force microscope (AFM, Bruker Icon) in tapping mode, with data planar fitting performed using NanoScope Analysis software. Chemical composition and molecular structure analysis were carried out using a confocal Raman spectroscopy system (WITec Alpha 300R), equipped with a triple grating spectrometer (1800 gr/mm) and a back-illuminated deep-cooled CCD detector. The experimental parameters were set as follows: a 532 nm solid-state laser (power 1 mW), a 100× oil immersion objective (NA=0.9, WD=0.3 mm), spectral acquisition range from 100 to 4000 $cm^{-1}$, integration time of 10 s/scan, and final data processing including baseline correction and Lorentzian fitting using WITec Project Plus software.

### Targeted Cleavage Specificity Profiling

Cas12a crRNA comprises two essential components: a universal scaffold region

(UAAUUUCUACUAAGUGUAGAU) for Cas12a protein recognition and binding, and a customizable region added to the 3’ end of the scaffold to confer specificity to the target sequence. The crRNA sequences were provided by Sangon Biotech (Shanghai) Co., Ltd. (see Table S2 for crRNA sequences). To verify the sequence-specific recognition ability of the crRNA, we established a Cas12a trans-cleavage detection system based on fluorescence resonance energy transfer (FRET). The standard reaction system (10 μL) consisted of the following components: 100 nM Cas12a nuclease (NEB, M0653T), 100 nM crRNA, 1× NEBuffer 2.1, 500 nM single-stranded fluorescence reporter probe (5’-6-FAM-TTATT-BHQ-1-3’, synthesized by Shenggong Biological), and a gradient concentration (0.1-100 nM) of double-stranded DNA target (dissolved in RNase-free water). After incubating the reaction mixture at 37 ℃ for 30 minutes, 10 μL of the product was subjected to 12 % native polyacrylamide gel electrophoresis (90 V, 1× TBE buffer, 2 hours). The target-dependent cleavage effect was double-validated through FAM fluorescence imaging (iBright FL 1500, excitation wavelength 488 nm) and SYBR Gold staining. Negative controls without crRNA and blank controls without target DNA were set up throughout the experiment to ensure the reliability of the results.

**PAGE Analysis**

In this study, native polyacrylamide gel electrophoresis (Native-PAGE) was used to characterize the "tetrahedral" DNA origami structure. The specific experimental procedure is as follows: First, an 8% separation gel system was prepared by sequentially adding 1 mL of 5× Tris-borate-EDTA (TBE) buffer, 2 mL of

acrylamide/bisacrylamide stock solution (29:1, w/w), 0.5 mL of 50% glycerol, 75 μL of 10% ammonium persulfate (APS), and 4 μL of tetramethylethylenediamine (TEMED) to 6.5 mL of ultrapure water. The solution was gently mixed for 30 seconds using a vortex mixer and immediately poured into the glass plates of a vertical electrophoresis apparatus (1.5 mm thickness). The gel was allowed to polymerize at room temperature, shielded from light, for 45 minutes to form a three-dimensional cross-linked network. After the gel had polymerized, the comb teeth were removed, and the sample wells were washed thoroughly with 1× TBE buffer to remove unpolymerized monomers. The gel plates were installed into the electrophoresis system (Bio-Rad), and the top and bottom chambers were filled with pre-chilled 1× TBE buffer. A total of 7 μL of DNA origami sample was mixed with 3 μL of 6× loading buffer and loaded precisely using a 10 μL micropipette, while DNA molecular weight markers were added to adjacent lanes. The electrophoresis system was powered on, set to a constant voltage of 90 V, and electrophoresis was carried out for 2 hours at 4℃ using a circulating water cooling system. After electrophoresis, the power was turned off, and the gel plates were removed and transferred to a staining box. The gel was stained with SYBR Gold nucleic acid dye (1× TBE dilution, v/v = 1:10,000) for 30 minutes, shielded from light. Finally, the gel image was captured using the iBright FL 1500 fluorescence imaging system.

**CdTe/ZnS QD-Conjugated DNA Origami**

The assembly efficacy between CdTe/ZnS QDs (QD-COOH, Xian Ruixi Biological

Technology Co., Ltd.) and DNA origami was achieved through EDC/NHS-mediated amide bond conjugation. The detailed procedure comprised three operational phases: Initially, 20 mM 1-ethyl-3-(3-dimethylaminopropyl)carbodiimide hydrochloride (EDC) and 10 mM N-hydroxysuccinimide (NHS) were dissolved in MES buffer (pH 6.0) and thoroughly mixed with CdTe/ZnS QD solution, followed by 1-hour oscillation incubation at 37℃ for carboxyl group activation. Subsequently, 5'-amino-modified CRISPR-$NH_2$ DNA strands (sequences listed in Table S1, synthesized by Sangon Biotech (Shanghai) Co., Ltd.) were introduced to facilitate directional DNA anchoring on QD surfaces, forming QD-DNA complexes. The reaction system underwent three consecutive centrifugation cycles (4℃, 10 min/cycle) with buffer replacement to remove uncoupled DNA strands, thereby establishing covalent conjugation via amide bonds between QD carboxyl groups and DNA amino terminals.

**S1**

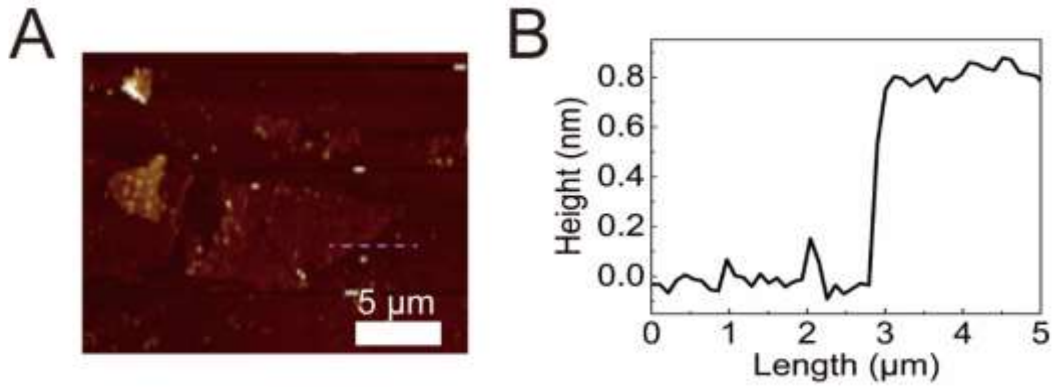


**Fig. S1. Characterization of mechanically exfoliated $MoS_2$ flakes.** (a) Three-dimensional AFM height mapping of $MoS_2$, showing surface topography of a typical mechanically exfoliated flake; (b) Height profile analysis along the dashed line in panel a revealing monolayer $MoS_2$ thickness of ~0.8 nm (scale bar: 5 μm).

**S2**

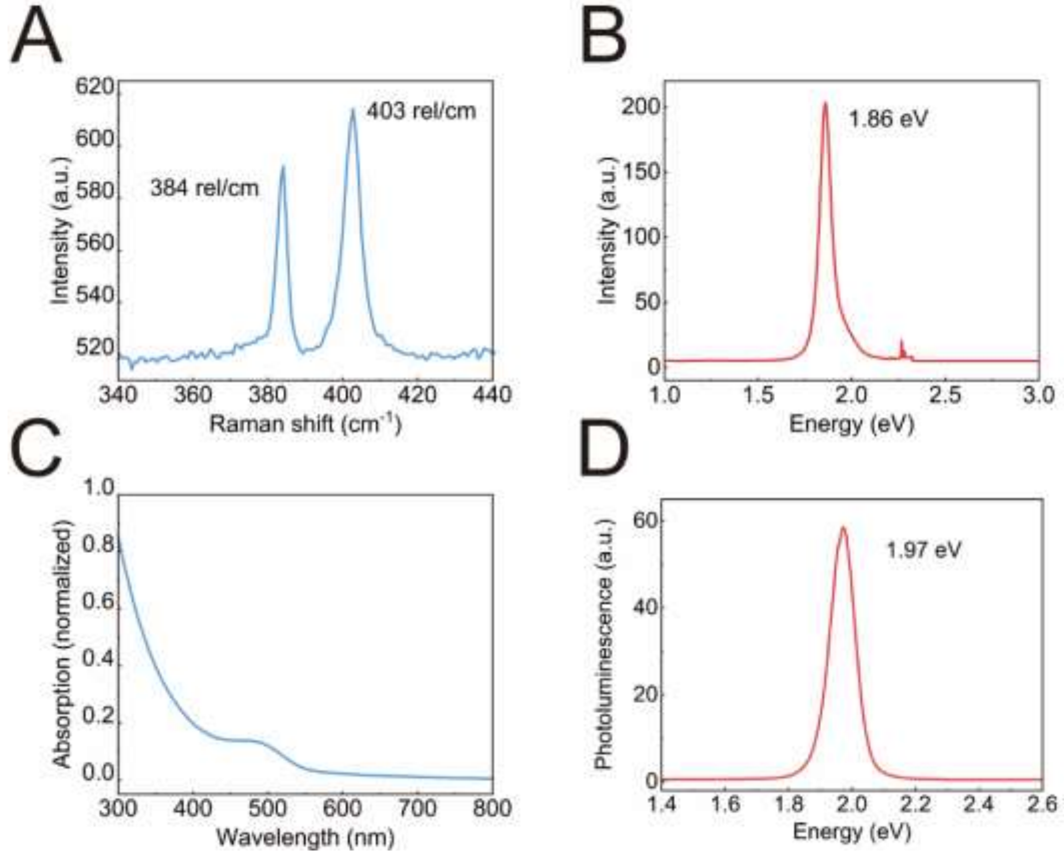


**Fig. S2. Spectroscopic characterization of 2D materials and QDs.** (a) Raman spectrum of mechanically exfoliated $MoS_2$ flake (532 nm excitation; (b) Photoluminescence (PL) spectrum of corresponding $MoS_2$ sample showing direct bandgap emission at ~1.86 eV; (c) UV-Vis absorption spectrum of CdTe/ZnS core-shell QDs; (d) Fluorescence emission spectrum of CdTe/ZnS QDs.

**S3**

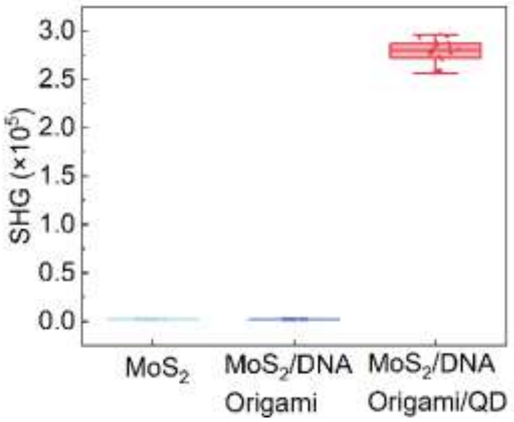


**Fig. S3. Statistical distribution validation of SHG spatial mapping data.** Box-whisker plot analysis of SHG intensity from 20 randomly selected measurement points within the central region of Figure 2(d).

**S4**

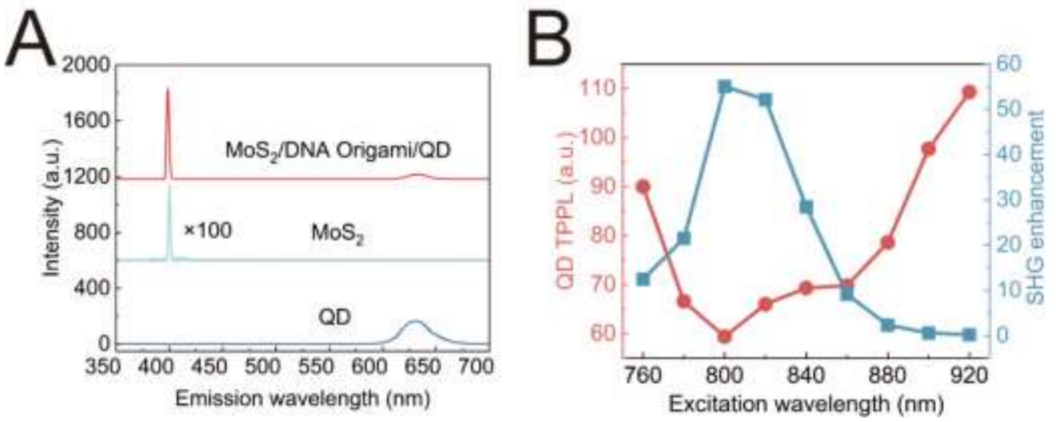


**Fig. S4. Nonlinear optical responses of hybrid materials under two-photon excitation.** (a) Comparative second harmonic generation spectra of pristine $MoS_2$ , QDs, and $MoS_2$ /DNA origami/QD hybrid system under 800 nm femtosecond pulsed laser excitation; (b) Wavelength-dependent two-photon absorption-induced photoluminescence (TPPL) intensity of pristine QD film (left axis, orange curve) and SHG enhancement factor of QD/$MoS_2$ hybrids in two-photon excitation regime (right axis, blue curve, normalized to SHG intensity of pristine $MoS_2$ ).

## S5

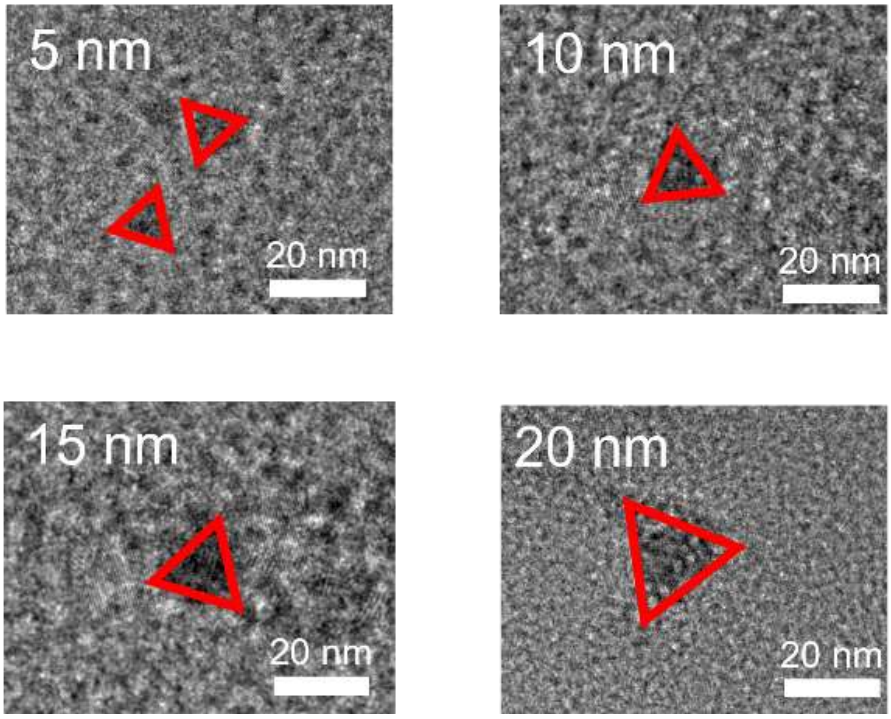


**Fig. S5. Size-controlled fabrication and morphological characterization of tetrahedral DNA origami nanostructures.** Transmission electron microscopy (TEM) images of tetrahedral DNA origami, negatively stained with 2% uranyl acetate. (scale bars: 20 nm).

## S6

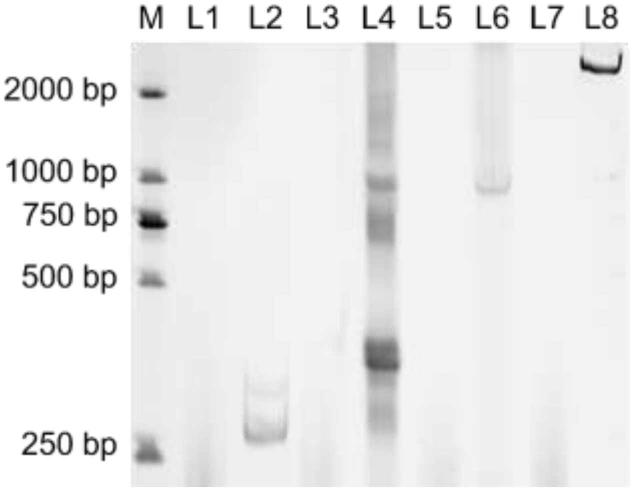


**Fig. S6. PAGE characterization of DNA origami probes with varying dimensions, where band mobility differences confirm structural integrity.** Polyacrylamide gel electrophoresis (PAGE) analysis demonstrated the size-dependent migration patterns of the origami structures (Fig. 4b). Prior to target binding, lanes 1 and 2 (5 nm origami), lanes 3 and 4 (10 nm origami), lanes 5 and 6 (15 nm origami) and lanes 7 and 8 (20 nm origami) exhibited distinct band positions corresponding to their molecular weights, respectively.

**S7**

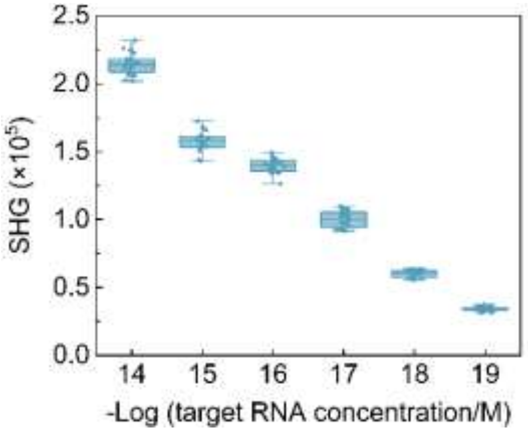


**Fig. S7. Statistical validation of SHG spatial mapping distribution.** Box-whisker plot analysis of SHG intensity from 20 randomly selected measurement points within the blue dashed box region of Fig. 4d.

**Table S1.**

| | | |
|---|---|---|
| 1 | 5nm-1 | NH2-CTTTACTCAACTTATTTTTTACATTCCTAAGTCTGAAACA TTACAGCTTGCTACACGAGAAGAGCCGCCATAGTA |
| 2 | 5nm-2 | NH2-TATCACCAGGCAGTTGACAGTGTAGCAAGCTGTAATAG ATGCGAGGGTCCAATAC |
| 3 | 5nm-3 | NH2-TCAACTGCCTGGTGATAAAACGACACTACGTGGGAATC TACTATGGCGGCTCTTC |
| 4 | 5nm-4 | NH2-TTCAGACTTAGGAATGTGCTTCCCACGTAGTGTCGTTTG TATTGGACCCTCGCAT |
| 5 | 10nm-1 | $NH_2$-CCCTGTACTGGCTAGGAATTCACGTTTTAATCTGGGCTTTGG GTTAAGAAACTCCCCG |
| 6 | 10nm-2 | CGCTGGAGGCGCATCACCGTTTGCGTATGTGTTCTGTGCGGCCT GCCGTCCCGTGTGGG |
| 7 | 10nm-3 | $NH_2$-CGGTGATGCGCCTCCAGCGCGGGGAGTTTCTTAACCCTTTC CGACTTACAAGAGCCGG |
| 8 | 10nm-4 | GCGAGACTCAGGTGGTGCCTTTGGCATTCGACCAGGAGATATCG CGTTCAGCTATGCCC |
| 9 | 10nm-5 | $NH_2$-CCCATGAGAATAATACCGCCGATTTACGTCAGTCCGGTTTCC CACACGGGACGGCAGGC |
| 10 | 10nm-6 | CGCACAGAACACATACGCTTTGGGCATAGCTGAACGCGATATCT CCTGGTCGAATGCC |
| 11 | 10nm-7 | $NH_2$-TTTTGCCCAGATTAAAACGTGAATTCCTAGCCAGTACAGGGT TTCCGGACTGACGTAAATCGG |
| 12 | 10nm-8 | CGGTATTATTCTCATGGGTTTGGCACCACCTGAGTCTCGCCCGG CTCTTGTAAGTCGG |
| 13 | 15nm-1 | AATGCTACTACTATTAGTAGAATTGATGCCACCTTTTCAGCTC GCGCCCCAAATGAAAATATAGCTAAACAGGTTA |
| 14 | 15nm-2 | TTGACCATTTGCGAAATGTATCTAATGGTCAAACTAAATCTAC TCGTTCGCAGAATTGGGAATC |
| 15 | 15nm-3 | AACTGTTATATGGAATGAAACTTCCAGACACCGTACTTTAGT TGCATATTTAAAACATGTTGAGCTAC |
| 16 | 15nm-4 | AGCATTATATTCAGCAATTAAGCTCTAAGCCATCCGCAAAAAT GACCTCTTATCAAAAGGAGCAATTAAAGGTACT |
| 17 | 15nm-5 | CTCTAATCCTGACCTGTTGGAGTTTGCTTCCGGTCTGGTTCG CTTTGAAGCTCGAATTAAAACGCGATATTTGAAGTCT |
| 18 | 15 nm-6 | TTCGGGCTTCCTCTTAATCTTTTTGATGCAATCCGCTTTGCTT CTGACTATAATAGTCAGGGTAAAGACCTGATTTTTG |
| 19 | 15nm-7 | ATTTATGGTCATTCTCGTTTTCTGAACTGTTTAAAGCATTTGA GGGGGATTCAATGAATATTTATGACGATTCCGCAGT |

| | | |
|---|---|---|
| 20 | 15nm-8 | ATTGGACGCTATCCAGTCTAAACATTTTACTATTACCCCCTCTGGCAAAACTTCTTTTGCAAAAGCCTCTC |
| 21 | 15nm-9 | CTTTACTCAACTTATTTTTTAGGGCCAATACTGCTTCGTCATA |
| 22 | 15nm-10 | TTTGACCATTAGATTAGACTGGATAGCGTGGTAATAGTAAAATGTTACATT |
| 23 | 15nm-11 | $NH_2$-TTTTCTGGTTCTGCGAACTTGATTTAG |
| 24 | 15nm-12 | CCAACAGGTCAGGAACAGTTGATTCCCAAAAGTTTCATTCCATATATTAGA |
| 25 | 15nm-13 | $NH_2$-TTTTGACTCGAACCAGACTTGCAAACT |
| 26 | 15nm-14 | AATATTCATTGAAAATTCGAGCTTCAAAGTCAAATATCGCGTTTTTCCCCC |
| 27 | 15nm-15 | GAGTACCTTTAATTTTTTCCTTTTGATTTAAGAGGAATTTTTCCGAAA |
| 28 | 15nm-16 | CATCAAAAAGAAAGAGGTCATTTTTGCGGATGAAGCGGATTG |
| 29 | 15nm-17 | AGAAGCAGCTTAGAGCTTAATTTTTTGAATATAATAAAGGTGGCATTTTTAATTCT |
| 30 | 15nm-18 | $NH_2$-TTTTACTATCTTTACCCTTTTTATAGTC |
| 31 | 15nm-19 | AATCAGGATAGTAGTAGCATTGAGAGGCGACCATAAATCAAA |
| 32 | 15nm-20 | TCAAATGCTTTAAATTCAGAAAACGAGAATTTTTGCAAAAGAAGTTTTTGCCAG |
| 33 | 15nm-21 | GCGCGAGCTGAGCTGTAGCTCAACATGTTTTATTCATTTGGG |
| 34 | 15nm-22 | TCGCAAATGGTCAATCTGTTTAGCTATATTAATATGCAACTAAAGTTTCGGTGT |
| 35 | 20nm-1 | AATGCTACTACTATTAGTAGAATTGATGCCACCTTTTCAGCTCGCGCCCCAAATGAAAATATAGCTAAACAGGTTATTG |
| 37 | 20nm-2 | ACCATTTGCGAAATGTATCTAATGGTCAAACTAAATCTACTCGTTCGCAGAATTGGGAATCAACTGTTATATGGAATGA |
| 38 | 20nm-3 | AACTTCCAGACACCGTACTTTAGTTGCATATTTAAAACATGTTGAGCTACAGCATTATATTCAGCAATTAAGCTCTAAG |
| 39 | 20nm-4 | CCATCCGCAAAAATGACCTCTTATCAAAAGGAGCAATTAAAGGTACTCTCTAATCCTGACCTGTTGGAGTTTGCTTCCG |
| 40 | 20nm-5 | GTCTGGTTCGCTTTGAAGCTCGAATTAAAACGCGATATTTGAAGTCTTTCGGGCTTCCTCTTAATCTTTTTGATGCAAT |
| 41 | 20nm-6 | CCGCTTTGCTTCTGACTATAATAGTCAGGGTAAAGACCTGATTTTTGATTTATGGTCATTCTCGTTTTCTGAACTGTTT |
| 42 | 20nm-7 | AAAGCATTTGAGGGGGATTCAATGAATATTTATGACGATTCCGCAGTATTGGACGCTATCCAGTCTAAACATTTTACT |
| 43 | 20nm-8 | ATTACCCCCTCTGGCAAAACTTCTTTTGCAAAAGCCTCTCGCTATTTTGGTTTTTATCGTCGTCTGGTAAACGAGGGTT |
| 44 | 20nm-9 | ATGATAGTGTTGCTCTTACTATGCCTCGTAATTCCTTTTGGCGTTATGTATCTGCATTAGTTGAATGTGGTATTCCTAA |
| 45 | 20nm-10 | ATCTCAACTGATGAATCTTTCTACCTGTAATAATGTTGTTCCG |

| | | |
|---|---|---|
| | | TTAGTTCGTTTT |
| 46 | 20nm-11 | ATTAACGTAGATTTTTCTTCCCAACGTCCTGACTGGTATAATG AGCCAGTTCTTAAAATCGCA |
| 47 | 20nm-12 | ACTGGCTCTTTTTTATACCAGTATTATTACAGTTAAGATTC |
| 48 | 20nm-13 | ACTAACGGAACAACCAGGACGTTGGGAAGAAAAATCTACGA ACAGTTG |
| 49 | 20nm-14 | ATTCCCAAAAGTTTCATTCCATATTTAATAAAACGA |
| 50 | 20nm-15 | $NH_2$-TTTTTGTCTGGTTCTGCGAACGAGTTTTTTAGTTTGACCA TTTCCTTT |
| 51 | 20nm-16 | TGATAAGATTTTGTCATTTTTGTAAATATGCATTAGTACGG |
| 52 | 20nm-17 | AGCTCAACATGTTTCGGATGGCTTAGAGCTTAATTGCTGACTA TTATA |
| 53 | 20nm-18 | GTCAGAAGCAGGTCTTTACCCTGAATATAATGCTGT |
| 54 | 20nm-19 | $NH_2$-TTTTCAAAAATCAAAGCGGATTGCATTTTCAA |
| 55 | 20nm-20 | AAAGATTACTGGATAGCTTTTTCCAATACTGCGAAAACGAGA TTCATAAAT |
| 56 | 20nm-21 | CTTTAAACAGTTCAGGAATCGTCATAAATATTCATTGAATACA TTCAA |
| 57 | 20nm-22 | CTAATGCAGAGATTTAGGAATACCCCCCCTCAAATG |
| 58 | 20nm-23 | $NH_2$-TTTTATCAGTTGATACATAACGCCATTTGAATTACGAGGC ATTTAAGA |
| 59 | 20nm-24 | AAAATGTTTAGAAGAGGAAGCCCGAAAGACTTGGGTAATAG T |
| 60 | 20nm-25 | TTTGCCAGAGGCAAATATCGCGTTTTAATTCGCAAAAGAAGT |
| 61 | 20nm-26 | CTTTACTCAACTTATTTTTTGCTTTTGAGCTTCAAAGCGAATT TTCAG |
| 62 | 20nm-27 | ACCGGAATTCATTTGGGTTTTTGCGAGCTGAAATAAAAACCA TTGCGAGAG |
| 63 | 20nm-28 | ACCAGACGACGAAGGTGGCATCAATTCTACTAACCCTCGTTT |
| 64 | 20nm-29 | TATCATAATAGTAGTAGCATTTGCGATTAGTAAGAGCAACAC |
| 65 | 20nm-30 | TTAGCTATATTGCAAACTCCAACAGGTCAGGAAATAACCTGT |
| 66 | 20nm-31 | CGCAAATGGTCTTAGAGAGTACCTTTAATTGCAGATACATTT |

**Table S1. Synthesis squence of DNA Origami (5 nm、10 nm、15 nm and 20 nm).**

**Table S2**

| | | |
|---|---|---|
| 1 | T-DNA$_{miRNA-21}$ | UAGCUUAUCAGACUGAUGUUGA |
| 2 | crRNA $_{miRNA-21}$ | UAAUUUCUACUAAGUGUAGAUUAGCUUAUCAG ACUGAUGUUGA |
| 3 | T-DNA$_{miRNA-155}$ | UUAAUGCUAAUCGUGAUAGGGGU |
| 4 | crRNA $_{miRNA-155}$ | UAAUUUCUAAGUGUAGAUGCAUUAAGGCUCCCG GAGCC |
| 5 | T-DNA$_{miRNA-10b}$ | UACCCUGUAGAACCGAAUUUGUG |

| | | |
|---|---|---|
| 6 | crRNA $_{\text{miRNA-10b}}$ | UAAUUUCUACUAAGUGUAGAUCGUCGCCGUCCAGCUCGACCAUUAUUAUUA |

**Table S2. Synthesis sequences for crRNA and target DNA.**
