# Supplementary material for: Ultrasensitive optoelectronic biosensor arrays based on twisted bilayer graphene superlattice
Source: Natl Sci Rev. 2025 Aug 23;12(10):nwaf357. doi: 10.1093/nsr/nwaf357 (PMC12492000; doi:10.1093/nsr/nwaf357)
Supplement: nwaf357_Supplemental_Files [file nwaf357_supplemental_files.zip › Teaser text.docx]

Teaser text

This study pioneers an opto-electronic biosensor by synergizing twist-engineered moiré superlattices in graphene with CRISPR technology, enabling sub-femtomolar nucleic acid detection under low-light conditions
